# Neuroinflammation of traumatic brain injury: Roles of extracellular vesicles

**DOI:** 10.3389/fimmu.2022.1088827

**Published:** 2023-01-18

**Authors:** Xilei Liu, Lan Zhang, Yiyao Cao, Haoran Jia, Xiaotian Li, Fanjian Li, Shu Zhang, Jianning Zhang

**Affiliations:** ^1^ Department of Urology, Tianjin Medical University General Hospital, Tianjin, China; ^2^ Tianjin Geriatrics Institute, Tianjin Medical University General Hospital, Tianjin, China; ^3^ Department of Neurosurgery, Tianjin Medical University General Hospital, Tianjin, China; ^4^ Tianjin Neurological Institute, Key Laboratory of Post-Neuroinjury Repair and Regeneration in Central Nervous System, Tianjin, China

**Keywords:** neuroinflammation, traumatic brain injury, extracellular vesicles, DAMP, TLRs, NLRP3, STING

## Abstract

Traumatic brain injury (TBI) is a major cause of neurological disorder or death, with a heavy burden on individuals and families. While sustained primary insult leads to damage, subsequent secondary events are considered key pathophysiological characteristics post-TBI, and the inflammatory response is a prominent contributor to the secondary cascade. Neuroinflammation is a multifaceted physiological response and exerts both positive and negative effects on TBI. Extracellular vesicles (EVs), as messengers for intercellular communication, are involved in biological and pathological processes in central nervous system (CNS) diseases and injuries. The number and characteristics of EVs and their cargo in the CNS and peripheral circulation undergo tremendous changes in response to TBI, and these EVs regulate neuroinflammatory reactions by activating prominent receptors on receptor cells or delivering pro- or anti-inflammatory cargo to receptor cells. The purpose of this review is to discuss the possible neuroinflammatory mechanisms of EVs and loading in the context of TBI. Furthermore, we summarize the potential role of diverse types of cell-derived EVs in inflammation following TBI.

## Introduction

Traumatic brain injury (TBI) is a severely disabling and deadly injury, with an estimated 69 million new cases each year (1). TBI is often classified as mild (GCS 13–15), moderate (GCS 9–12), or severe (GCS <9) according to the Glasgow Coma Scale (GCS). Moderate and severe TBI is a leading cause of neurological disability and loss of health in young adults in all countries and represents a major burden to families and social economies (2, 3). Mild TBI (mTBI, commonly known as concussion) was recognized in the past as an inessential injury. However, there has been gradual awareness of the relationship between mTBI and neurodegenerative diseases such as Alzheimer’s disease (AD), Parkinson’s disease (PD), frontotemporal dementia (FTD), and chronic traumatic encephalopathy (CTE) (4–7). In addition, TBI can be categorized into primary injury and secondary injury on the basis of the pathological process. Mechanical forces (acceleration/deceleration, rotation, and direct or penetrating force) acting on the head or skull immediately lead to primary injury, such as skull fracture, cerebral contusion, cranial hematoma, and axonal injury (8, 9).

A series of secondary events subsequent to the initial insult comprising excitotoxicity caused by the neurotransmitter glutamate, free radical generation, and the neuroinflammatory response (10–12) develops within minutes and persists from hours to months after the primary insult that damages nerve cells, glia, and blood vessels (13). It is well established that these secondary injuries are crucial determinants of outcomes, especially TBI-associated neuroinflammation (14). In the acute phase of TBI, the initial injury results in the death of brain cells along with release of damage-associated molecular patterns (DAMPs). DAMPs bind to recipient cells, initiating immune responses and releasing pro-inflammatory mediators. In addition, activation of microglia and astrocytes, as well as infiltration of peripheral immune cells, further enhances inflammatory responses (15). All of these factors create a pro-inflammatory environment that is closely related to tissue damage and poor neurological outcomes (16).

Recently, extracellular vesicles (EVs), which are released by all major cells, are being recognized as greatly important for cell-to-cell communication. Many studies have shown that EVs exert vital effects on biological and pathological processes in the central nervous system (CNS), including neuronal differentiation, synaptic formation, glial crosstalk, and regulation of homeostatic signaling and the immune system (17–19). Moreover, it is well known that EVs are key mediators of CNS neuroimmune interactions and serve as essential regulators of neuroinflammation following TBI by carrying various kinds of cargo, such as DNA fragments, RNA, lipids, and proteins (20, 21). In this review, we summarize the pro-inflammatory function of EVs and the overall effect of diverse cell type-derived EVs on TBI-associated neuroinflammation.

## Traumatic brain injury and neuroinflammation

Neuronal inflammation is involved in a wide range of processes with essential roles in all aspects of physiology and pathology in the CNS. Neuroinflammation is a complex physiological response that is of important value for clearance of pathogens and the regeneration of damaged brain tissue. However, excessive and uncontrollable inflammation can result in autoimmune disorders and tissue damage (22). Within minutes post-TBI, a robust sterile immune response develops, which is characterized by danger signals released by neurons and glia, activation of resident innate immune cells, recruitment and infiltration of peripheral immune cells, and secretion of multiple inflammatory molecules (23). Nerve cells, meninges, and the blood–brain barrier (BBB) are damaged by the initial insult at the site of the injury and the amount of DAMPs released, such as high-mobility group box 1 protein (HMGB1), adenosine triphosphate (ATP), heat shock proteins (HSPs), and uric acid (13, 24, 25). These DAMPs interact with receptors, including Toll-like receptors (TLRs) and nucleotide-binding oligomerization domain (NOD)-like receptors (NLRs), to trigger innate immune responses (26, 27). TLR4, which is an extensively studied TLR, exerts crucial effects on neuroinflammatory responses in CNS diseases and injuries. DAMPs bind to TLR4, and activated TLR4 initiates activation of myeloid differentiation factor 88 (MyD88). A series of pathways, including nuclear factor κ-light chain-enhancer of activated B cells (NF-κB) and mitogen-activated protein kinase (MAPK), is subsequently triggered, and a diverse variety of chemokines and cytokines are released that amplify the inflammatory response (28, 29). In addition to TLRs, DAMPs bind with various NLRs. Among these, nucleotide oligomerization-like receptor protein 3 (NLRP3) has been extensively studied in TBI. The NLRP3 inflammasome comprises three protein domains: the sensor protein NLRP3, the apoptosis-associated speck-like protein adapter (ASC), and the precursor enzyme pro-caspase-1 (30). TBI induces activation of the NLRP3 inflammasome in neurons, astrocytes, and microglia in the cortex, and activated caspase-1 promotes cleavage of pro-interleukin (IL)-1β, pro-IL-18, and the amino terminus of gasdermin-D (GSDMD) (31). Cleaved IL-1β, IL-18, and GSDMD ultimately cause cell pyroptosis, which is accompanied by the release of pro-inflammatory cytokines (32, 33). Numerous studies have demonstrated that knockout or pharmacologic inhibition of the NLRP3 inflammasome can alleviate neuroinflammation and improve neurological outcomes after brain injury (34–36). Moreover, purinergic receptor signaling and the complement system contribute to the development of neuroinflammation (37–40).

Recent studies have suggested that the cyclic GMP-AMP synthase (cGAS) and stimulator of interferon genes (STING) pathways promote the production and release of pro-inflammatory cytokines to further aggravate TBI-associated neuroinflammation (41, 42). The concentration of cytosolic mitochondrial DNA (mtDNA), a kind of mitochondrial DAMP, is elevated in the pericontusional cortex after TBI. cGAS is able to bind mtDNA and activate STING and downstream transcription factors, leading to the production of type I interferon (IFN-I)-related genes (41). Knockout of cGAS or STING improves histological measures and neurological outcomes after brain injury (41).

In the acute stage of TBI, resident microglia usually respond by rapidly migrating to the site of the initial insult (23). Activated microglia polarize into two main states: classically activated M1 microglia and alternatively activated M2 microglia (15). M1 microglia play an important role in the clearance of pathogens and cell debris. However, M1 microglia release pro-inflammatory mediators that aggravate the inflammatory response and tissue damage. In contrast, M2 microglia promote tissue repair and neurological function recovery through the secretion of anti-inflammatory molecules (43, 44). Nevertheless, the definition of “M1/M2 microglial polarization” is controversial and uncertain, and intermediate activation states may exist (45). Astrocytes are widely distributed in the CNS and perform a great diversity of essential functions. Astrocytes shift toward astrogliosis and are called “reactive astrocytes” after brain injury (46). Several investigations have shown that reactive astrocytes adopt an inflammatory phenotype after CNS injuries and diseases (47–49), and activation of the NF-κB pathway is a central step in the pro-inflammatory role of reactive astrocytes in CNS diseases (50–52). Pro-inflammatory mediators, including IL-1β, IL-17, tumor necrosis factor (TNF)-α, IL-6, and reactive oxygen species (ROS), and sphingolipids such as sphingosine 1-phosphate (S1P) act as promoters that trigger nuclear translocation of NF-κB in astrocytes to enhance neuroinflammation (53–55). TBI also induces recruitment and infiltration of peripheral immune cells by releasing cytokines, chemokines, and purines (15). Neutrophils enter the brain parenchyma at the early stage after cortical injury (peak 24–48 h) (56). Subsequently, macrophages and lymphocytes migrate into the core of the injury site (57). These peripheral immune cells aggravate the inflammatory response by releasing cytokines, chemokines, and ROS (58).

Secretion of cytokines by diverse cells in the CNS exerts pro-inflammatory effects on TBI (59). IL-1β and TNF-α, as traditional pro-inflammatory cytokines, are elevated after TBI in humans and animals (60–62). ROS activate redox-regulated transcription factors, such as MAPKs, NF-κB, and activator protein 1 (AP-1), many of which are involved in the inflammatory process (63). IL-17 is a crucial pro-inflammatory factor that can lead to neuroinflammation and neuronal apoptosis (64). IL-6 is also known to be a contributor to neuroinflammation, and elevated levels of IL-6 in cerebrospinal fluid are associated with poor outcomes following TBI (65). Inflammasome activation leads to the release of IL-1β and IL-18 during sterile inflammatory responses (33). In addition, IFN-I-related genes are significantly elevated in an aged TBI model and are considered to be key factors in trauma-associated neuroinflammation and neurodegeneration (42).

## Extracellular vesicles

EVs were initially considered to be debris and useless components of cells. However, there is developing awareness of the biological functions of EVs in various pathological and physiological processes (66). EVs are categorized into multiple types, exosomes, microvesicles (MVs) or microparticles (MPs), and apoptotic bodies, based on their size and formation mechanism. Exosomes, with a length of 30–150 nm, are released from cells *via* fusion of multivesicular endosomes with the plasma membrane. MVs are 50–1,000 nm in size and are formed by direct outward blebbing of the cell membrane, as are apoptotic bodies, which are more than 1,000 nm in size (67). EVs exist in almost all bodily fluids, such as blood, tissue fluid, or urine, and a number of studies have shown that EVs are generated and activated by diverse stimuli in diseases, such as oxidative stress, inflammatory responses, hypoxia, aging, cell death, and exposure to radiation (68–73). In fact, exosomes and MVs are known messengers that facilitate intercellular communication by carrying and delivering distinct biomolecules, including nucleic acids (DNA and multiple RNA types), proteins, lipids, metabolites, and organelles (17, 74). EVs are taken up by target cells, ultimately leading to biological changes in the recipient cells (75).

Accumulating evidence has emphasized the important role of EVs in biological and pathological processes such as the regulation of neuronal development and synaptic plasticity, maintenance of CNS homeostasis, propagation and removal of neurotoxic proteins, modulation of the immune system, and neuroinflammation in CNS diseases (76). Cerebral endothelial cell-derived EVs improve the reconstruction of synaptic function by upregulating the expression of miRNA-126-3p after ischemia/reperfusion brain injury (77). Astrocyte-derived EVs promote neuronal synapse formation and enhance the survival and electrophysiological function of neurons (78, 79). In AD, brain-derived extracellular vesicles (BDEVs) propagate Tau pathology in GABAergic interneurons and disrupt lipid metabolism (80, 81). In contrast, Aβ-EVs released by microglia move at the neuron surface and impair synaptic plasticity (82). Moreover, microglia-derived EVs loaded with cytokines lead to the progression of the neuroinflammatory response and systemic immune responses after brain injury (83). Thus, evidence to date suggests the value of EVs and their cargo on intercellular interactions in CNS health and diseases.

## E (EVs) and traumatic brain injury

**Figure 1:**
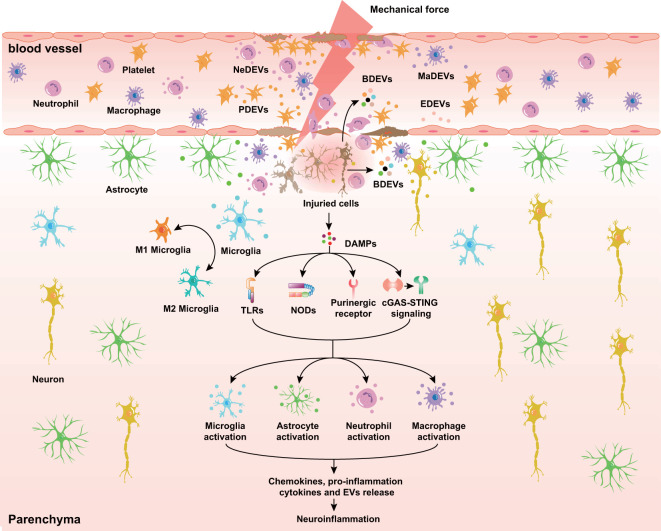
Diverse EVs released in response to TBI. At the acute stage of TBI, BDEVs, including neuron-, microglia cell-, and astrocyte-derived EVs, are released into the lesion area and peripheral circulation through the disrupted BBB. Endothelial cells, immune cells (neutrophils and macrophages), and platelets release EVs in response to mechanical force and secondary injuries. Additionally, DAMPs released from dying neurons interact with prominent receptors such as TLRs, NODs, and purinergic receptors and initiate the secretion of cytokines, chemokines, and EVs in microglia, astrocytes, and immune cells. NeDEVs, Neutrophil-derived extracellular vesicles; BDEVs, Brain derived extracellular vesicles; PDEVs, Platelet-derived extracellular vesicles; MaDEVs, Macrophage-derived extracellular vesicles; EDEVs, Endothelial cell-derived extracellular vesicles; DAMPs, Damage associated molecular patterns; BBB, Blood-brain barrier; TLRs, Toll-like receptors; NODs, Nucleotide-binding oligomerization domains.

Numerous studies have suggested that not only the primary insult but also secondary injuries of TBI can influence the number and characteristics of EVs (**Figure 1**). For example, in response to mechanical injury, brain endothelial cells release EVs enriched with tight junction protein occludin when undergoing vascular remodeling (84). Oxidative stress and ROS increase the release of EVs that regulate inflammation and vascular calcification (85–87). Interestingly, lipopolysaccharide (LPS), ATP, and pro-inflammatory factors such as IL-1β, TNF-α, and IFN-γ activate microglia and promote the release of EVs encapsulating a distinct profile of cargo (e.g., pro-inflammatory cytokines, miRNAs, and proteins) (88–90), indicating the crucial role of EVs derived from microglia in neuroinflammation. In addition, TBI and neuroinflammation induce the release of astrocyte-derived EVs containing neurotoxic complement proteins and miRNAs. Similarly, the increased level of miR-21 in neuron-derived EVs may contribute to neuroinflammation after TBI (20).

In addition to local release, EVs are found in other biological fluids after TBI. For example, the concentration of EVs in serum is elevated rapidly in TBI patients (91, 92). Moreover, another study reported that the concentration of EVs in cerebrovenous blood is higher than that in arterial blood, indicating an elevated number of circulating EVs derived from the brain (93). Similarly, an increase in the number of EVs after TBI has been found in rodent studies (94). BDEVs comprise glial fibrillary acidic protein-positive (GFAP^+^) and neuron-specific enolase-positive (NSE^+^) EVs and reach peak levels at 3 h post-TBI. The number of GFAP^+^ EVs decreased at 6 h post-TBI, while NSE^+^ EVs persisted consistently at the same time (94). The injured brain also releases EVs into the cerebrospinal fluid (CSF), the concentration of which is approximately 2-fold greater than that in control CSF (95). Interestingly, TBI induces inflammatory changes in salivary-derived EVs, showing that EVs are a potential mediator of cell-to-cell signaling.

Given that EVs can cross the BBB and are detectable in the CSF and peripheral circulation after TBI, EVs are valuable potential biomarkers that have drawn increasing attention (96). EVs represent an abundant source of information from parent cells, and identification of specific disease signatures may be possible by analyzing their cargo. A recent study concluded that EVs in military persons with mTBI have higher levels of Tau, amyloid-beta (Aβ) 42, and IL-10, which are associated with chronic postconcussive symptoms and neuroinflammation (97). Phosphorylated Tau (p-Tau), neurofilament light (NfL), IL-6, and TNF-α are also increased in CNS-enriched EVs in older veterans and are associated with cognitive impairment and inflammation (98). Moreover, miRNAs in circulating EVs are significantly altered in mTBI patients; for example, the expressions of hsa-miR-139-5p and hsa-miR-18a-5p, which correlate with inflammation and CNS diseases, are markedly different (99). In addition, TBI induces changes in the protein composition of EVs in the CSF. Several protein biomarkers, such as αII-spectrin breakdown products (BDPs), GFAP, and presynaptic terminal protein synaptophysin, are present in CSF EVs (95). Another study has suggested that the neuroregeneration-related protein flotillin-1 is only present in the CSF after TBI and that downregulation of ADP-ribosylation factor 6 (Arf6) and delayed upregulation of Ras-related protein Rab7a (Rab7a) in the CSF are related to unfavorable outcomes post-TBI (100). Interestingly, inflammatory gene expression in salivary-derived EVs is altered after TBI, indicating that salivary-derived EVs may become biomarkers for evaluating the severity of TBI (101). Overall, EVs and their cargo in the CNS and peripheral circulation undergo tremendous changes in response to TBI, and EVs in biological fluids are desirable biomarkers for the diagnosis and evaluation of TBI.

## Extracellular vesicles (EVs) and TBI-associated neuroinflammation

**Figure 2:**
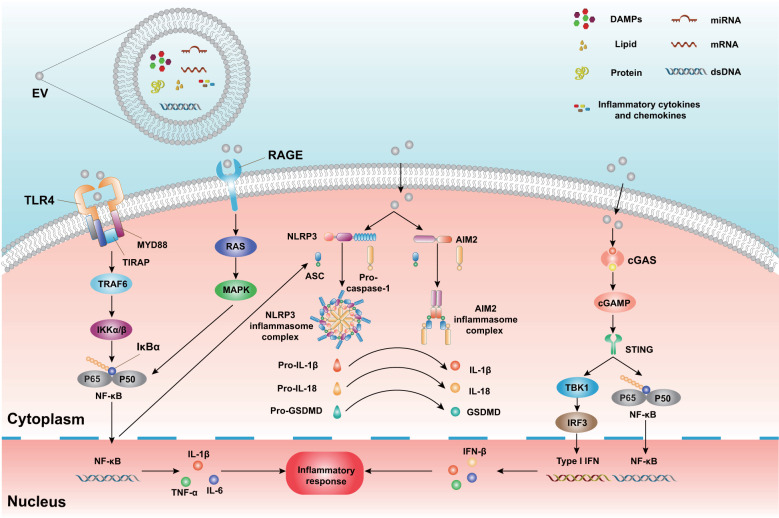
The mechanism of neuroinflammatory modulation by EVs and cargo. EVs carry distinct biomolecules, including nucleic acids (DNA and multiple RNA types), proteins, lipids, DAMPs, cytokines, and chemokines. These cargoes interact with relevant receptors and ultimately lead to an inflammatory response in recipient cells. EVs bind to TLRs and initiate the activation of the downstream signaling proteins MyD88, TIRAP, TRAF6, and IκBα. In addition, EVs bind to RAGE and activate the Ras and MAPK pathways. These pathways subsequently trigger the activation of downstream NF-κB and generation of pro-inflammatory cytokines (IL-1β, TNF-α, IL-6). During TBI, EVs interact with NLRP3 and AIM2 inflammasomes and promote the activation of caspase-1. Activated caspase-1 cleaves pro-IL-1β, pro-IL-18, and pro-GSDMD, resulting in pyroptosis and pro-inflammatory molecule release. Additionally, EVs release and carry dsDNA post-TBI. These EVs bind to cGAS and activate cGAMP and STING. Subsequently, STING triggers the TBK1, IRF3, and NF-κB pathways and ultimately leads to IFN-I transcription and inflammatory mediator release. DAMPs, Damage associated molecular patterns; TLRs, Toll-like receptors; MyD88, Myeloid differentiation factor 88; TIRPAP, Toll-interleukin 1 receptor domain-containing adaptor protein; TRAF6, TNF receptor-associated factor 6; IκBα, inhibitor of NF-kB; RAGE, The receptor for advanced glycation end products; MAPK, Mitogen-activated protein kinase; NF-κB, Nuclear factor κ-light chain-enhancer of activated B cells; NLRP3, Nucleotide oligomerization-like receptor protein 3; AIM2, absent in melanoma 2; GSDMD, Gasdermin-D; cGAS, the cyclic GMP-AMP synthase; cGAMP, cyclic dinucleotide (CDN)-2′,3′-cyclic guanosine monophosphate-adenosine monophosphate; STING, stimulator of interferon genes; TBK1, TANK-binding kinase 1; IRF3, Interferon regulatory factor 3; IFN-I, type I interferon.

CNS-resident and peripheral immune cells trigger sterile immune reactions quickly in response to TBI. Previous studies have demonstrated that as a novel mechanism, EVs participate in neuroimmune and neuroinflammatory responses. Below, we briefly describe the important role of EVs in TBI-induced sterile immune reactions and the subsequent inflammatory cascade (**Figure 2**).

## EV-derived Damage associated molecular patterns (DAMPs), toll-like receptors, and The receptor for advanced glycation end products (RAGE) in traumatic brain injury

TLRs are pattern recognition receptors (PRRs) able to recognize pathogens and DAMPs released from injured and dead cells, followed by activation of the immune response and expression of inflammatory genes. Ten functional TLRs are expressed in humans (TLR1–TLR10); 12 functional TLRs are expressed in mice (TLR1–TLR9, TLR11–TLR13) (102). Elevated expression of TLRs and activation of downstream signaling enhancing the inflammatory cascade have been reported in experimental studies of TBI. A previous study reported that TLR1, TLR2, and TLR4 mRNA are increased in the pericontusional area at 24 h after TBI (103). Moreover, protein expression levels of TLR2 and TLR4 in damaged tissue peak at 24 h to 7 days and decrease at 14 days post-TBI (104, 105). TLR2 and TLR4 are known to contribute to intensifying neuroinflammation after TBI. For instance, TLR2 knockout inhibits the activation of microglia and astrocytes, decreases the secretion of inflammatory cytokines, and alleviates secondary injuries caused by TBI (106, 107). Furthermore, the expression of TLR4, phospho-NF-κB p65 (p-p65), and MyD88 is elevated after TBI, which leads to the release of pro-inflammatory cytokines (108). TLR4 knockdown suppresses autophagy and astrocyte activation and decreases the level of pro-inflammatory cytokines (109), and TLR4 knockout improves microglial polarization from the M1 phenotype toward the M2 phenotype, which inhibits the inflammatory response after TBI (110).

EVs have been shown to possess the ability to carry DAMPs such as HMGB1, S100 proteins, HSPs, ATP, and histones, which bind to TLRs to enhance the inflammatory response (111). Among them, HMGB1, which has been most extensively studied, is released passively from necrotic neurons following TBI or is packaged in EVs (112, 113). HMGB1 binds to TLRs and/or advanced glycation end products (RAGE) and initiates the activation of the MyD88-dependent signal transduction cascade, including MyD88, Toll-interleukin 1 receptor domain-containing adaptor protein (TIRAP), TNF receptor-associated factor 6 (TRAF6), and inhibitor of NF-κB (IκBα), subsequently triggering the activation of downstream NF-κB and the MAPK pathway and leading to the release of pro-inflammatory cytokines. One study reported that treatment with an HMGB1 antagonist blocks HMGB1 release, attenuates the expression of TLR4, MyD88, and p65, and promotes neurological function recovery in a TBI model (114). Inhibition of caspase-1 inhibits the inflammatory activity of the HMGB1/TLR4/NF-κB pathway in TBI mice (115). In addition, treatment with glycyrrhizin, which has been suggested to inhibit HMGB1, decreases pro-inflammatory release in damaged sites of TBI by inhibiting the HMGB1/RAGE pathway (116). Interestingly, the HMGB1-RAGE axis has been found to be involved in the pulmonary dysfunction induced by TBI (117). Indeed, several studies have suggested that S100 proteins, HSPs, and ATP are involved in neuroinflammation mediated by the TLR4 or RAGE pathway (118–120).

## EVs and the nucleotide oligomerization-like receptor protein 3 inflammasome/absent in melanoma 2 inflammasome in traumatic brain injury

In addition to TLRs, NLRs, including NLRP3 and absent in melanoma 2 (AIM2)-like receptors, are other PRRs that interact with DAMPs to initiate innate immune responses and subsequently neuroinflammatory responses after TBI. The NLRP3 inflammasome comprises three protein domains: the sensor protein NLRP3, ASC, and pro-caspase-1. The NLRP3 inflammasome is triggered by multiple stimuli and sequentially results in the activation of caspase-1. Then, IL-1β and IL-18 are cleaved by caspase-1 from their inactive pro-isomers to their active forms. Caspase-1 cleaves GSDMD, which produces pores on the cell membrane and terminally induces the release of IL-1β and IL-18. Similarly, the AIM2 inflammasome contains a pyrin domain (PYD) and a hematopoietic, interferon-inducible, and nuclear (HIN) domain. Activation of the AIM2 inflammasome leads to cleavage of pro-IL-1β, pro-IL-18, and pro-GSDMD and pro-inflammatory molecule release. Several studies have revealed that NLRP3 and AIM2 participate in the neuroinflammatory response post-TBI. The NLRP3 inflammasome is expressed in the cerebral cortex after TBI and activates caspase-1, IL-1β, and IL-18 in a rat model (31). Moreover, knockout or pharmacologic inhibition of the NLRP3 inflammasome can alleviate neuroinflammation and improve neurological outcomes after brain injury (34, 36). The AIM2 inflammasome is activated in neurons by the CSF after TBI and mediates neuronal cell death (121). The AIM2 inflammasome also induces brain microvascular endothelial cell pyroptosis and exacerbates BBB damage post-TBI (27). Notably, the AIM2 inflammasome is involved in the pathological process of TBI-induced acute lung injury (ALI) (122).

EVs are released into the serum after TBI, which contains inflammasome proteins and promotes the activation of a neural-respiratory-inflammasome axis, resulting in an inflammatory response in the lung (122). Furthermore, the number of EVs and levels of ASC are evidently elevated in TBI patients. *In vitro*, these EVs increase inflammasome activation and mediate lung human microvascular endothelial cell pyroptosis (92). Treatment with enoxaparin, which blocks EV uptake, mitigates inflammasome activation in the brain and lungs after injury (123). EVs can also activate the TLR4/MyD88/NF-κB pathway by delivering DAMPs and initiating NLRP3 inflammasome activation at the transcriptional level. EVs can carry ROS, which is considered to trigger activation of the NLRP3 inflammasome (124, 125).

## EVs and the cyclic GMP-AMP synthase/stimulator of interferon genes (cGAS/STING) pathway in traumatic brain injury

The cGAS-STING pathway plays a crucial role in neuroinflammatory responses in TBI (41, 42). cGAS binds to double-stranded DNA (dsDNA) arising from DNA released by damaged and dead cells and EVs carrying DNA and mtDNA (126). Then, STING is activated by cyclic dinucleotide (CDN)-2′,3′-cyclic guanosine monophosphate-adenosine monophosphate (cGAMP), which is synthesized by activated cGAS. Subsequently, STING triggers TANK-binding kinase 1 (TBK1), which promotes the phosphorylation of IFN regulatory factor 3 (IRF3) and NF-κB and ultimately leads to IFN-I transcription and inflammatory mediator release (41, 126).

EVs release and carry the DNA sensor STING, playing a role in antiviral effects during herpes simplex virus 1 infection (127). Oxidized DNA released from EVs activates the PARP1-cGAS-NF-κB pathway and worsens the inflammatory pathology (128). Furthermore, EVs carrying dsDNA trigger STING activation and aggravate inflammation. Administration of GW4869, an antagonist of EV release, restores EV-induced STING signaling activation and the inflammatory response (129). Overall, EVs may become a promising target in neuroinflammation triggered by cGAS-STING signaling after TBI.

## Extracellular vesicles and immune cell activation in traumatic brain injury

In general, activation of resident innate immune cells (e.g., microglia and astrocytes) and recruitment of peripheral immune cells (e.g., leukocytes and macrophages) result in the release of various cytokines and chemokines and intense inflammation. Considering that EVs transfer bioactive factors from parent cells to recipient cells and activate diverse signaling pathways in the latter to influence their function, EVs, as mediators of cell communication, are crucially important in the neuroinflammatory response after TBI. BDEVs are released into the extracellular space and increase microglia/macrophage activation and pro-inflammatory cytokine release, promoting inflammation. Lactadherin, also known as milk fat globule-EGF factor-8 (MFGE-8), promotes clearance of BDEVs and alleviates neurological deficits (130, 131). Moreover, von Willebrand factor (vWF)-bound BDEVs improve vascular leakage and systemic coagulation after TBI (132), and EVs loaded with miRNAs that mediate immune cell interactions have been described. For example, neuron-derived EVs carrying miR-21-5p and miR-9-5p promote M1 microglia polarization *in vitro* and *in vivo* to aggravate neuroinflammation (133, 134). Astrocyte-derived EVs enriched with miR-873a-5p inhibit the transformation of M1 microglia and decrease the activation of the ERK and NF-κB pathways (135). In addition, mast cell-derived EVs containing miR-409-3p improve microglial activation and inflammation by activating the NF-κB pathway (136).

Cytokine release plays a critical role in neuroinflammation in CNS injuries and diseases. Numerous studies have demonstrated that EVs are carriers of cytokines. EVs released from LPS-stimulated microglia express diverse pro-inflammatory factors, such as IL-1β, TNF-α, IL-6, C-C Motif Chemokine Ligand 2 (CCL2), and Nitric oxide synthase 2 (NOS2), *in vitro* (83). Similarly, another study found that concentrations of TNF and IL-6 are significantly increased in EVs from LPS-stimulated microglia (88). Manganese activates microglia, which propagate ASC and IL-1β by releasing EVs (137). Additionally, hydrostatic pressure increases the concentration of pro-inflammatory mediators in microglia-derived EVs (138), and circulating EVs in players with sport-related concussion express high levels of IL-6 compared with uninjured players (139). Taken together, EVs are involved in immune cell activation and inflammatory dissemination *via* bioactive compounds such as miRNAs and cytokines. EVs, released by almost all kinds of cells, targetrecipient cell and exert pro- or anti-inflammatoryeffects after TBI. Herein, we systematicallyreviewed the roles of diverse cell-derivedextracellular vesicles and their cargoes in regulatingneuroinflammation after TBI (**Table 1**).

**Table 1:** Role of diverse EVs and their cargoes in TBI and inflammation condition.

EV origins	Stimulus/Model	EVs cargoes	EVs function	References
Primary microglia	ATP	Proteins implicated in cell adhesion/extracellular matrix organization, autophagy-lysosomal pathway, and cellular metabolism	Activate recipient astrocytes	(140)
BV2 microglia	rTBI brain extracts	miR-124-3p	Inhibit neuronal inflammation and contribute to neurite outgrowth	(141)
BV2 microglia	IL-4	miR-135a-5p	Downregulated the expression of NLRP3 and inhibited the apoptosis of neuronal cells	(142)
Blood and CD11b-isolated microglia/macrophages	TBI model	IL-1β and miR-155	Activate microglia *in vitro* and initiate neuroinflammation following intracortical injection in naive animals	(83)
Primary astrocytes	/	/	Ameliorated oxidative stress by activating Nrf2/HO-1 signaling in the hippocampus of TBI rats	(143)
Human brain astrocytes	/	lncRNA (NKILA)	Alleviate neuronal injury by binding to miR-195 and upregulating NLRX1 in TBI	(144)
Primary astrocytes	/	GJA1-20 k	Downregulate the apoptosis rate and upregulate mitochondrial function to promote neuronal recovery.	(145)
Primary astrocytes	/	miR-873a-5p	Inhibit LPS-induced microglial M1 phenotype transformation and the subsequent inflammation	(135)
Brain	TBI/Stroke model	/	Activate microglia/macrophage and amplify neuroinflammation	(130) (131),
Plasma	TBI patients	C3b and C5b-9 terminal C complex	Activate immune response	(146)
Plasma and primary astrocytes	IL-1β	Protein and microRNA	promote the transmigration of leukocytes through modulation of the peripheral acute cytokine response	(147)
HT-22	TNF-α	miR-34a and miR-146a	Induce mitochondrial dysfunction in a neuronal cell type	(148)
Brain	TBI model	miR-21	activate microglia and promote inflammation	(20)
PC-12	/	miR-21-5p	Cause polarization of M1 microglia and aggravate neuroinflammation	(133)
Neutrophils	LPS	miR-142–3p and miR-451	Trigger an inflammatory cascade and induce direct vascular damage	(149)
Neutrophils	Migrating	miR-1260, miR-1285, miR-4454, and miR-7975	Promote polarization of pro-inflammatory macrophage	(150)
Platelet	Trauma hemorrhage	Histone H4	Induce platelet ballooning and microparticle release	(151)
Platelet	extracellular mitochondria from TBI	/	Produce ATP and ROS, which promote platelet activation and PDEV release	(151)
Platelet	/	Mitochondrial DAMPs	Activate inflammation response	(152)
Platelet	/	IL-1β and caspase-1	Promote inflammasome activation and contribute to thromboinflammation	(153)
Plasma	LPS	miR-155	Induce systemic inflammation	(154)
Serum	LPS or partial hepatectomy	/	Induce neuroinflammation	(155)
Plasma	Mechanically ventilated	IL-1β, caspase-1, and gasdermin D	Cause neuroinflammation	(156)

ATP, Adenosine triphosphate; rTBI, repetitive traumatic brain injury; NLRP3, Nucleotide oligomerization-like receptor protein 3; Nrf2, Nuclear factor erythroid 2-related factor 2; HO-1, Hemeoxygenase-1; NLRX1, Nucleotide binding and oligomerization domain (NOD)-like receptor X1; GJA1-20k, gap junction protein alpha 1 truncated monomer-20k; C3b, Complement 3b; C5b-9, Complement 5b-9; LPS, Lipopolysaccharide; ROS, Reactive oxy gen species; PDEV, Platelet-derived extracellular vesicles.

## Diverse cell-derived extracellular vesicles and neuroinflammation in traumatic brain injury

### Microglia/macrophage-derived extracellular vesicles

Microglia are resident immune cells and continuously monitor the environment in the CNS and rapidly respond to changes. Microglia perform functions such as removing cell debris, phagocytosing necrotic cells and pathogens, releasing various cytokines and chemokines that regulate the inflammatory response, promoting tissue repair, and maintaining brain homeostasis (157, 158). After the onset of TBI, resident microglia usually respond instantly to the site of the initial insult (23). Then, microglia become transformed from the resting state to the activated state. Activated microglia are traditionally polarized into two main subtypes: M1 and M2 microglial phenotypes (15). However, some studies consider that a number of intermediates exist, as opposed to binary divisions of activated microglia such as M1 and M2. M1-phenotype microglia possess the ability to clear pathogens and cell debris as well as pro-inflammatory mediators that aggravate the inflammatory response and tissue damage. In contrast, M2 microglia exert an important role in tissue repair and neurological function recovery by suppressing neuroinflammation (43, 44).

In recent years, EVs released from activated microglia have been seen as an essential factor in the regulation of neuroinflammation. During neuroinflammation, microglia release larger-size EVs than in the healthy condition, and the concentrations of IL-6 and TNF are simultaneously markedly elevated in these EVs. In addition, the TNF signaling pathway is of significance in the production of microglia-derived EVs (88). Furthermore, extracellular ATP plays an important role in microglia-derived EV release. ATP promotes the secretion of EVs containing IL-1β mRNA from microglial cells upon neuroinflammation and modifies the proteome of these EVs (140, 159). Upon ATP stimulation, P2X7R triggers the p38 MAPK signaling pathway and subsequently activates acid sphingomyelinase, which ultimately leads to EV shedding and IL-1β release (160). Blockade of P2X7R has been reported to decrease the number of MDEVs in the pericontusional cerebral cortex (161).

MDEVs are a double-edged sword in neuroinflammation. On the one hand, treatment with MDEVs improves the activation of autophagy and decreases the expression of neuroinflammation and inflammasome signaling pathways in microglia to maintain homeostasis in the CNS (162). MiR-124-3p increases significantly in MDEVs after TBI, which promotes M2 microglial polarization. EV-derived miR-124-3p suppresses neuronal inflammation and promotes neurite outgrowth by targeting PDE4B and inhibiting the mTOR signaling pathway (141). Another study reported that MDEVs containing miR-124 promote resting microglia toward the M2 phenotype and enhance neurogenesis by inhibiting the TLR4 pathway after TBI. Moreover, normal MDEVs increase the expression of pro-regeneration-related markers such as IL-10 and TGF-β in astrocytes (140). EVs derived from M2 microglia containing miR-135a-5p alleviate brain injury by downregulating the Thioredoxin-interacting protein (TXNIP/NLRP3) axis (142). On the other hand, microglia-derived EVs are detected in the circulation after TBI, and circulating EVs post-TBI are sufficient to activate microglia. Activated MDEVs package IL-1β and miR-155, which can initiate the neuroinflammatory response following injection into the cerebral cortex (83). MDEVs also increase the production of pro-inflammatory molecules and ROS and improve the phagocytic efficiency and proliferation of microglia (138). Membrane-associated Low-density lipoprotein (LDL) receptor-related protein-1 shedding from microglia aggravates and sustains inflammation (163), and EVs derived from microglia can influence astrocytes and change the state of astrocytes. Indeed, MDEVs trigger the pro-inflammatory activity of astrocytes by transferring IL-1β mRNA (159). Microglia-derived EVs upregulate both the pro-inflammatory factor IL-6a and the neuroinflammatory factor IL-10 in astrocytes (140). EV release from microglia carrying diverse proteins may influence the response of recipient astrocytes (140). In summary, these studies highlight the key role of MDEVs in the neuroinflammatory response after TBI and implicate their contribution to delivering pro-inflammatory information to astrocytes.

### Astrocyte-derived extracellular vesicles

Astrocytes are widely distributed in the CNS and perform a series of important functions, including synapse formation, axonal transmission and myelination, restriction of oxidative stress and inflammation, and regulation of BBB permeability (164). In addition to their functions in modulating CNS homoeostasis, astrocytes have a crucial role in neuroinflammation by switching to reactive astrocytes. Reactive astrocytes not only generate alarmins or DAMPs but also express TLR4 and RAGE, which trigger NF-κB transduction and lead to a pro-inflammatory cytokine release, such as IL-1β, TNF-α, cyclooxygenase-2 (COX-2), and matrix metalloproteinase 9 (MMP-9), to amplify neuroinflammation (165, 166). During CNS infections, injuries, and diseases, astrocytes switch to an inflammatory phenotype and may contribute to subsequent inflammatory responses (47). In contrast, reactive astrocytes contribute to removing debris from the injured brain and forming glial scars to ameliorate neuroinflammation (167). Additionally, TGF-β, nerve growth factor, and brain-derived neurotrophic factor, which exert anti-inflammatory roles in TBI, are released by astrocytes (168).

Similar to the mechanism by which EVs are released from microglia, TNF-α treatment elevates glutaminase expression and increases astrocyte-derived extracellular vesicle (ADEV) release under neuroinflammatory conditions. Furthermore, ATP is released into the extracellular space by mechanical force after TBI and activates the P2X7R and p38 MAPK pathways, followed by activation of acid sphingomyelinase and EVs released from astrocytes (160). Emerging studies have demonstrated the neuroprotective role of ADEVs. For instance, ADEVs activate the Nrf2/HO-1 signaling pathway to protect the survival of neurons by inhibiting mitochondrial oxidative stress after TBI (143). ADEVs also promote neuronal recovery by delivering the long noncoding RNA NKILA and gap junction alpha 1-20 k (144, 145). Of note, a high expression of miR-873a-5p in ADEVs alleviates microglia-induced neuroinflammation by suppressing microglial M1 pro-inflammatory phenotype polarization and downregulating the phosphorylation of ERK and NF-κB p65 after TBI (135). Regarding the pro-inflammatory role of ADEVs in TBI, BDEVs comprise ADEVs (GFAP^+^) and neuron-derived EVs (NSE^+^) that possess the ability to activate microglia/macrophages and amplify neuroinflammation after TBI and stroke (130, 131). Moreover, plasma ADEVs carry neurotoxic complement proteins such as complement 3b (C3b) and the C5b-9 terminal C complex, indicating that ADEVs may be involved in the immune response (146). Pro-inflammatory cytokines stimulate ADEV release and alter the cargo of ADEVs, which may modulate the neuroinflammatory response (169). Interestingly, EVs released by IL-1β-stimulated astrocytes cross the BBB and improve the migration of leukocytes in the brain. These EVs inhibit peroxisome proliferator-activated receptor α (PPARα) and increase the activation of the NF-κB signaling pathway, which triggers cytokine production in the liver (147).

### Neuron-derived extracellular vesicles

Following severe TBI, neuronal cells die in two aspects: initial mechanical damage and secondary injuries such as oxidative stress and neuroinflammation. DAMPs are released from dying neuronal cells and activate pro-inflammatory M1 microglia that contribute to the neuroinflammatory response (170). Moreover, TBI leads to substance P release by neurons, which augments the inflammatory response by promoting glial cell activation, degranulation of mast cells, and leukocyte migration (9).

The important role of neuron-derived extracellular vesicles (NDEVs) in neuroinflammation has been identified. For instance, NDEVs in Gulf War illness patients contain higher concentrations of HMGB1 and pro-inflammatory cytokines such as IL-1β, IL-6, and TNF-α than those from healthy controls, indicating that NDEVs are involved in the persistence or dissemination of neuroinflammation (171). In an *in vitro* experiment, EVs derived from young neurons suppressed astrocytic reactivity and inhibited inflammation (172). Cortical NDEVs containing miR-181c-3p can downregulate CXCL1 in astrocytes and alleviate neuroinflammation after ischemic brain injury (173). NDEVs transfer miR-124-3p to microglia and astrocytes and exert a protective role against spinal cord injury (174). In addition, neurons transfer pathological α-synuclein to astrocytes *via* NDEVs and cause inflammation (175). NDEVs carrying miR-9-5p polarize microglia toward the M1 subtype and exacerbate neuroinflammation (134). In TBI, miR-34a and miR-146a are increased in NDEVs and cause mitochondrial oxygen consumption and ROS production in recipient cells when neurons are exposed to inflammation (148). MiR‐21 is primarily expressed in neurons near the lesion site surrounding activated microglia (20), and NDEVs carrying miR-21-5p are phagocytosed by microglia and cause polarization of M1 microglia, which aggravates neuroinflammation (133).

### Neutrophil-derived extracellular vesicles

Neutrophil-derived extracellular vesicles (NeDEVs) can be classified into two subtypes, NeDEVs and neutrophil-derived trails (NeDTRs), according to the mechanism of their generation. NeDEVs are generated not only spontaneously from neutrophils but also in response to bacteria and bacterial products, cytokines and chemokines, and complement components (176). Interestingly, NeDEVs exert either pro-inflammatory or anti-inflammatory actions depending on diverse conditions. Resting neutrophils produce EVs that can reduce the generation of ROS and cytokine release from neutrophils to mitigate inflammation. In contrast, activated neutrophil-derived EVs release ROS and various cytokines that aggravate the inflammatory response (177). NeDEVs transfer miR-142–3p and miR-451 to endothelial cells and lead to endothelial inflammation (149), and neutrophils interact with platelets and initiate the innate immune response by directly transmitting NeDEVs (178). Recently, NeDTRs were identified as another subtype of neutrophil-derived EVs generated from migrating neutrophils. During migration, integrin-mediated physical forces stretch the uropods of neutrophils, resulting in the detachment of tail protein and release of NeDTRs. NeDTRs carry pro-inflammatory miRNAs such as miR-1260, miR-1285, miR-4454, and miR-7975 that promote polarization of pro-inflammatory macrophages (150).

### Platelet-derived extracellular vesicles

Platelets exert key physiological effects on hemostasis and coagulation, but recently, the unique role of platelets and platelet-derived extracellular vesicles (PDEVs) in immunity and inflammation in many injuries and diseases, such as arthritis, tendon injuries, epilepsy, acute lung injury, preeclampsia, and vasculitis, has been well described (153, 179–182). TBI causes trauma hemorrhage, and circulating platelets participate in hemostasis through transformation into procoagulant balloons. Ballooning platelets carry histone H4 and lead to healthy platelet activation as well as the release of large numbers of EVs (151). Extracellular mitochondria (exMT) bind to platelets and produce ATP and ROS, which promote platelet activation and PDEV release after TBI (183). PDEVs convey DAMPs such as HMGB1 and mtDNA, which activate TLRs and aggravate inflammation (152, 184). PDEVs activate neutrophils and macrophages *via* C-Type Lectin DomainContaining 5A (CLEC5A) and TLR2, promoting neutrophil extracellular trap formation and pro-inflammatory factor secretion in platelet–leukocyte interactions (185). Moreover, IL-1β- and caspase-1-decorated PDEVs promote inflammasome activation and contribute to thromboinflammation through purinergic signaling (153, 184).

### Circulating cell-derived extracellular vesicles

As mentioned above, the number and characteristics of EVs are altered after TBI. Moreover, BDEVs, including MDEVs, ADEVs, and NDEVs, can cross through the damaged BBB and be released into the circulation. EVs carrying bioactive molecules that contribute to the systemic immune response induced by TBI have been reported (16). Recently, emerging evidence suggests that circulating EVs influence neuroinflammation. LPS-stimulated serum-derived EVs promote the activation of microglia and astrocytes and mRNA expression of pro-inflammatory factors and miR-155 (154). Intracerebroventricular injection of pro-inflammatory EVs causes the release of pro-inflammatory cytokines in the CSF, and these EVs intravenously injected have the ability to cross the BBB and induce neuroinflammation (155). EVs from the plasma of hyperammonemic rats show altered protein cargo, which is primarily related to immune system processes. Injection of these EVs aggravates neuroinflammation by activating glial cells and the NF-κB signaling pathway and releasing pro-inflammatory cytokines (186). Circulating EVs from ALI lead to the activation of microglia and the pyroptosis pathway, which worsen neuroinflammation (156). Collectively, these studies show that circulating EVs play a crucial role in neuroinflammation.

## Concluding remarks and future perspectives

The accumulating evidence regarding EV cargo and biological functions has well described their role in neuroinflammation. In this review, we summarized that EVs, as a unique mechanism, mediate immunity and neuroinflammation through cell-to-cell communication among various cells in the CNS and circulation, as well as the underlying role of diverse cell-derived EVs in TBI-associated neuroinflammation. Although these findings provide a new perspective on the potential mechanism of TBI-associated neuroinflammation, we are still far from thoroughly understanding the precise mechanisms of EVs in neuroinflammation. First, isolation and purification of EVs from specific cells and purification techniques should be improved and refined. Second, the biological functions of large EVs (e.g., microvesicles) and small EVs (e.g., exosomes) in the pathogenesis of neuroinflammation are complex and controversial, and the pro-inflammatory or anti-inflammatory effects of these EVs need further study and explanation. Third, it is unclear whether inhibition of the biogenesis, secretion, and uptake of pro-inflammatory EVs or promotion of their removal is favorable for treating TBI-associated neuroinflammation.

EVs have promising potential for guiding the field of clinical diagnosis and therapy post-TBI. Biomarkers of EVs can be helpful in determining the progression and prognosis of TBI. In addition, EVs have the potential to be applied in advance personalized medicine approaches for neuroinflammation therapy by accurately delivering various biological materials or anti-inflammatory drugs to the insult region. With our awareness of the crucial roles that EVs play in neuroinflammation after TBI, there are many opportunities for us to drive this field into new areas such as TBI and neurodegeneration.

In conclusion, EV biology is a prospective field that largely deepens our understanding of pathological processes in health and disease. Further investigation of the clinical application of EVs might be a promising therapeutic strategy for neuroinflammatory and neurodegenerative diseases post-TBI.

## Author contributions

All authors contributed to the study’s conception and design. XLL searched the bibliography and drafted the manuscript. LZ and YC prepared the figures. HJ, FL and XTL prepared the table. SZ and JZ critically revised the manuscript. All authors contributed to the article and approved the submitted version.
